# Proposition of New Testing Procedure for the Mechanical Properties of Bulk-Fill Materials

**DOI:** 10.3390/ma16134868

**Published:** 2023-07-07

**Authors:** Matea Macan, Ana Marošević, Bruno Špiljak, Luka Šimunović, Matej Par, Danijela Marovic, Danijela Juric-Kacunic, Zrinka Tarle

**Affiliations:** 1 Department of Endodontics and Restorative Dental Medicine, School of Dental Medicine, University of Zagreb, 10000 Zagreb, Croatia; mmacan@sfzg.hr (M.M.); amarosevi@sfzg.hr (A.M.); bruno.spiljak@gmail.com (B.Š.); mpar@sfzg.hr (M.P.); 2 Department of Orthodontics, School of Dental Medicine, University of Zagreb, 10000 Zagreb, Croatia; lsimunovic@sfzg.hr; 3 Private Dental Clinic, 76571 Gaggenau, Germany; danijela.juric@zahnarztpraxis-juric-gaggenau.de

**Keywords:** bulk-fill composites, degree of conversion, mechanical properties, high-intensity light-curing polymerisation, artificial aging, thermal cycling, testing method

## Abstract

This study analysed flexural properties, microhardness, and the degree of conversion (DC) of five bulk-fill composites under clinically relevant conditions (4 mm thick specimens) in comparison to 2 mm specimens according to ISO 4049. Additionally, the effect of rapid polymerisation on 4 mm specimens was evaluated after accelerated aging. DC was measured using Fourier transform infrared spectrometry at 2 and 4 mm thick layers, while flexural properties and Vickers microhardness were tested using 16 × 2 × 2 mm or 16 × 2 × 4 mm specimens. Three polymerisation protocols were used: (I) “ISO”: 2 mm thickness, 1000 mW/cm^2^, double-sided; (II) “10 s”: 4 mm thickness, 1000 mW/cm^2^, one-sided; and (III) “3 s”: 4 mm thickness, 2600 mW/cm^2^, one-sided. Mechanical properties were tested after 1 day, after 10,000 thermocycles, and after 10,000 thermocycles followed by a 7-day immersion in absolute ethanol. The “ISO” protocol produced a higher DC and microhardness of all materials. Elastic modulus was significantly higher for the “ISO” protocol compared to the 4 mm specimens. The differences in flexural strength for all polymerisation protocols were equalised after thermocycling and immersion in absolute ethanol. All tested materials met the ISO 4049 flexural strength requirement (80 MPa) for all polymerisation methods and all aging conditions. Rapid polymerisation achieved nearly optimal properties (ISO), except for elastic modulus, which was significantly reduced in 4 mm samples.

## 1. Introduction

In everyday clinical dentistry, precision and efficiency are valued, but there is also an increasing emphasis on speed. Bulk-fill composite materials are used for direct dental restorations to reduce the time of a dental visit. One of the most important features of bulk-fill composite materials is that they can be applied in 4 to 5 mm thick layers, resulting in a shorter and simpler clinical procedure [1]. Longitudinal clinical studies show that there is no negative impact on the quality of fillings compared to the conventional 2 mm layering when using conventional composites [2,3].

To enable such use of the material, it was necessary to change its composition [4]. Some manufacturers have switched to using filler volume fractions and larger filler particles, and thus correspondingly, a smaller filler surface area, which provides less light scattering and better light transmission through the material [5,6]. Instead of radically modifying the chemical composition of the material, some manufacturers seem to simply reduce the amount of pigment and use larger filler particles to improve translucency [7].

In contrast, some manufacturers significantly changed the composition of the material. For example, Tetric PowerFill (Ivoclar Vivadent, Schaan, Liechtenstein, PFL) [8,9] uses addition fragmentation chain transfer (AFCT) technology. The AFCT reagent β-allyl sulfone is responsible for stimulating the so-called stepwise polymerisation, which leads to the formation of shorter polymer chains [9]. Another manufacturer uses two special types of monomers to reduce polymerisation shrinkage and stress in Filtek One Bulk Fill Restorative (3M, St. Paul, MN, USA; FIL): addition–fragmentation monomers and very high-molecular weight monomers with fewer reactive sites per unit of volume [10]. An interesting approach to achieve a higher degree of conversion (DC) was taken with Tetric PowerFill and its low-viscosity counterpart, Tetric PowerFlow (Ivoclar Vivadent, Schaan, Liechtenstein, PFW). Both materials show an increase in opacity (decrease in translucency) during polymerisation. In the unpolymerised state, the blue light transmission of Tetric PowerFlow is approximately 28%, which allows light penetration and the initiation of polymerisation at a depth of 4 mm. After polymerisation, Tetric PowerFlow shows lower translucency, less than 10%, similar to dentin [8].

A significant reduction in polymerisation time (to 3 s) of methacrylate-based composites was only achieved when a conventional Norrish type II photoinitiator system consisting of camphorquinone and tertiary amine was replaced by Norrish type I photoinitiators (e.g., monoacylphosphine oxide) used under suitable polymerisation conditions (>500 mW/cm^2^, wavelength range 395–415 nm) [11,12]. In contrast to the Norrish type II photoinitiators, which form one free radical per molecule of the photoinitiator, the Norrish type I photoinitiator is homolytically decomposed upon irradiation with violet light, and forms two free radicals [12]. These changes in the composition of the composites required the modification in the output spectrum of LED polymerisation devices, i.e., the addition of violet light, which was achieved in the third and fourth generation of LED polymerisation devices.

In addition to the abovementioned changes in composition, Tetric PowerFill and Tetric PowerFlow bulk-fill composites also contain a special photoinitiator, bis-(4-methoxybenzoyl) diethylgermanium. This is a Norrish type I germanium-based photoinitiator, known commercially as Ivocerin. It allows a greater depth of polymerisation, shortens the polymerisation time and is more efficient than camphorquinone [12].

In order to mimic the conditions in the oral cavity and to evaluate the tendency of dental restorative materials to degrade, it is necessary to perform some of the procedures of artificial ageing. Specimens may be exposed to demineralised water or artificial saliva for a longer period of time or placed in ethanol or enzyme solutions for a shorter period of time. Artificial ageing of the material can also be achieved by the thermocycling process [13].

Thermocycling is an in vitro process in which materials are exposed to a temperature range similar to that in the oral cavity. It is based on the diffusion of heat and moisture, in the case of porous specimens [14]. Simulated changes in temperature and storage media can critically affect the overall condition of the bulk-fill material and consequently alter its mechanical properties and morphological characteristics. Exposure to 10,000 cycles between 5 °C and 55 °C has been found to be equivalent to approximately one year of clinical use in human oral cavity [14]. 

The use of ethanol-based solutions or other organic solvents is associated with a deterioration of the mechanical properties [15]. Ethanol and dimethacrylate monomers used in most composites have similar solubility parameters. Ethanol therefore easily penetrates the material, causing the plasticisation of the resin and the deterioration of the mechanical properties [15,16,17]. 

According to ISO 4049, the 2 mm thickness is defined for the specimens used for measuring flexural strength (FS) and flexural modulus (FM) [18], which is appropriate for conventional composites. However, when using bulk-fill materials, dentists can apply a layer of material with a thickness of 4 mm. In addition, only one side of the material is exposed to the curing light, in contrast to the ISO 4049 protocol, which recommends polymerising each specimen on both sides. In our previous study, we used two 2 mm thick stacked specimens separated by the PET film and polymerised only on one side to mimic the 4 mm layer thickness [4]. It was found that the material behaves differently at a depth of 2–4 mm. Therefore, the material properties measured with ISO 4049 do not seem to be valid for the entire thickness of the restoration. The main drawback of the mentioned study was the fact that the transparent film most likely hindered the polymerisation reaction in the lower 2–4 mm because the activated radicals could not initiate polymerisation in the lower parts of the sample and the chain lengthening could not progress in the lower parts. El-Askary et al. investigated the FS of bulk-fill materials with a specimen thickness of 4 mm and a distance of 2 or 8 mm from the light-curing device [19]. Interestingly, they found that the FS was significantly higher for thicker samples cured at a distance of 8 mm from the light source than that of the 2 mm thick samples [19]. This suggests that light irradiation affects the FS of thick composite specimens more than thin 2 mm specimens. Based on these results, we wanted to investigate the influence of different polymerisation protocols on selected bulk-fill composites at the maximum allowable specimen thickness of 4 mm, which corresponds to clinical conditions.

The aim of this study was to evaluate the influence of a customised testing protocol on bulk-fill composites under clinically relevant conditions (4 mm specimen thickness and one-sided polymerisation) in comparison to the control group prepared according to the ISO 4049 protocol. In addition, the influence of rapid polymerisation with very high light intensity and accelerated ageing was evaluated. The investigated properties were: DC, FS, FM, and microhardness (MH).

The null hypotheses were:

1. There is no difference between 2 mm specimens (polymerised according to the ISO standard) and 4 mm specimens (polymerised according to the rapid 3 s or 10 s protocol).
2. There is no difference in the investigated properties when comparing different polymerisation protocols.
3. There is no difference in FS, FM, or MH after 24 h, 10,000 thermocycles, and 10,000 thermocycles followed by 7 days of immersion in absolute ethanol.
4. There is no difference among the materials in the investigated properties.

## 2. Materials and Methods

### 2.1. Materials

Five bulk-fill composite materials were tested, of which three were high-viscosity materials and two were low-viscosity materials, as listed in Table 1.

### 2.2. Testing Methods

Polymerisation was initiated using a fourth generation LED curing unit (PowerCure, Ivoclar Vivadent AG; Schaan, Liechtenstein). Three polymerisation protocols were used:

1. 3 s protocol: during 3 s with an average value of 2600 mW/cm^2^;
2. 10 s protocol: during 10 s with an average value of 1000 mW/cm^2^;
3. ISO protocol: during 20 s with an average value of 1000 mW/cm^2^.

**Figure 1:**
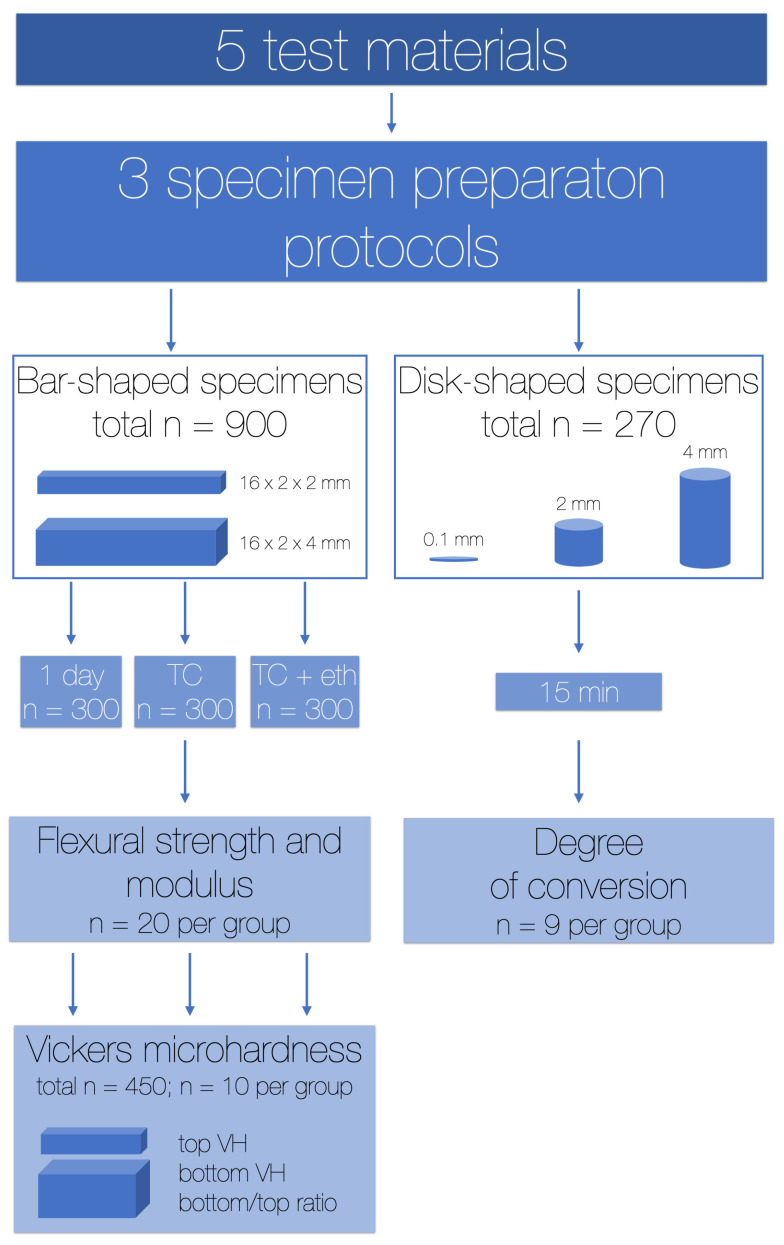
Flowchart of the experiments. TC—thermocycling; eth—absolute ethanol.

The following tests were performed, as depicted in Figure 1:

1. Three-point bending test: FS and FM (2 and 4 mm, short-term and after accelerated ageing);
2. MH (2 and 4 mm, short-term and after accelerated ageing);
3. Degree of conversion (0.1 and 2 or 4 mm).

#### 2.2.1. Specimens for Three-Point Bending Test and Microhardness

A hundred and eighty specimens per each material were made. Specimens with dimensions 16 × 2 × 2 mm and 16 × 2 × 4 mm were prepared in the Teflon moulds. The split-moulds were positioned on a glass base and had a Teflon frame holding the two parts together that ensured the dimensional stability. The upper and lower surfaces of the mould were covered with polyethylene terephthalate (PET) foil. The material was placed in the mould using the OptraSculpt instrument (Ivoclar Vivadent AG; Schaan, Liechtenstein).

Twenty specimens were made for each material, specimen preparation protocol and aging were performed, making a total of 900 specimens (20 × 5 × 3 × 3), divided into three groups:

1. “3 s” group—4 mm thick specimens polymerised 3 times for 3 s with radiant exitance of 2600 mW/cm^2^ only on one side (3 times in total);
2. “10 s” group—4 mm thick specimens polymerised 3 times for 10 s with radiant exitance of 1000 mW/cm^2^ only on one side (3 times in total);
3. “ISO group”—2 mm thick specimens polymerised according to ISO 4049 [18]—3 times for 20 s with radiant exitance of 1000 mW/cm^2^ on each side (6 times in total).

After polymerisation, each specimen was manually ground with sandpaper (silicon carbide grinding paper, Grit500/P1000, Buehler; Lake Bluff, Illinois, USA) for the purpose of removing excess material and assuring the uniformity of dimensions of all the specimens. The dimensions of the specimens were checked using a digital calliper (Alpha Professional Tools; Franklin, NJ, USA).

After preparation, the specimens were stored in plastic containers filled with 5 mL of distilled water in the dark and in an incubator, at 37 °C.

##### Measurement Time Points/Aging

Three measurement time points were defined:

- One day in distilled water (n = 20);
- Thermocycling (t) (n = 20);
- Thermocycling and 7 days in absolute alcohol (tA) (n = 20).

##### Thermocycling Protocol

Two-thirds of the specimens (n = 600) after preparation were stored in distilled water for 21 days in the dark, i.e., in an incubator (37 °C) and were then subjected to a thermocycling process (Thermocycler, SD Mechatronik; Feldkirchen-Westerham, Germany).

Thermocycling consisted of 10,000 cycles with a dwell time of 30 s at temperature from 5–55 °C. After thermocycling, half of these specimens (n = 300) were subjected to mechanical properties testing, while the rest (n = 300) were placed in absolute ethanol for 7 days, after which further testing was performed.

##### Three-Point Bending Test

All specimens were subjected to a three-point bending test on a universal testing device (Inspekt Duo 5 kN-M, Hegewald & Peschke, Meß- und Prüftechnik GmbH, Nossen, Germany) at a crosshead speed of 1 mm/min. Flexural strength was calculated according to the formula:

$$\sigma =\frac{3FL}{2bh^{2}}\;[\mathrm{MPa}]$$

where ***F*** represents the maximum force; ***L*** is the distance between support points; ***b*** is the width; and ***h*** is the height of the specimen.

The flexural modulus was calculated according to the formula:

$$E=\frac{FL^{3}}{4bh^{3}d}\;[GPa]$$

where ***d*** represents the deflection of the specimen under the load ***F***.

##### Microhardness Testing

MH was measured with a Vickers hardness tester (ESI Prüftechnik GmbH; Germany) on specimens previously used in the three-point bending test, with n = 10 per experimental group. The measurements were performed with a 100 g load and 15 s dwell time at five spots of each specimen, on the top and bottom surfaces (2 or 4 mm) and the mean value of five repetitions was calculated for each specimen. The top surface of the specimens was the one adjacent to the light-cured unit or, in the case of ISO, the one that was light-cured first. The top surfaces were marked when the specimens were fabricated. The lower surface was considered to be the surface opposite the upper surface.

The hardness tester diamond left an imprint of the shape of a pyramid in the material. Using a light microscope, the diagonals (***d*_1_**, ***d*_2_**) of the base of the pyramid were measured, and the hardness was determined according to the formula:

$$HV=F\frac{0.189}{d^{2}}$$

where ***F*** is the applied force in N and ***d*** is the arithmetic mean value of the diagonals of the base of the pyramid in mm:

$$d=\frac{d_{1}+d_{2}}{2}$$

The bottom/top ratio was calculated for each specimen by dividing the HV value on the bottom by the HV value on the top.

#### 2.2.2. Degree of Conversion 

DC was measured by attenuated total reflectance Fourier transform infrared (ATR-FTIR) spectroscopy using the iS50 spectrometer (Thermo Fisher Scientific Inc.; Madison, WI, USA) 15 min after polymerisation. 

Uncured composites (n = 6) were placed in custom-made silicone moulds (d = 2, h = 0.1, 2 or 4 mm) and light-curing was performed over the PET foil covering the top surface of the specimen. 

The 0.1 mm specimens were polymerised by the 3 previously mentioned polymerisation protocols: 3 s (3 s with radiant exitance of 2600 mW/cm^2^), 10 s (10 s with radiant exitance of 1000 mW/cm^2^), or ISO protocol (20 s with radiant exitance of 1000 mW/cm^2^). Specimens with a height of 2 mm were polymerised by the ISO protocol for 20 s, while specimens with a height of 4 mm were polymerised by the 3 s or 10 s protocol.

FTIR spectra were captured 15 min post-curing. The DC was calculated by comparing the relative change in integrated intensities of the band at 1638 cm^−1^ (aliphatic C=C bonds) and the reference band at 1608 cm^−1^ (aromatic C⦁⦁⦁C bonds), according to the following formula:

$$DC\;(\%)=\left(1-\frac{R_{polymerised}}{R_{unpolymerised}}\right)\times 100$$

where R is defined as:

$$R=\frac{aliphatic\;C=C\;integratedintensity}{aromatic\;C⦁⦁⦁C\;integratedintensity}$$

### 2.3. Statistical Analysis

The required sample size was estimated by power analysis using data collected in a preliminary study. The desired difference to be detected at a significance level of 0.05 was as follows: 5% for degree of conversion, 15% for flexural strength and modulus, and 15% for microhardness. The power analysis was performed using G*Power version 3.1 (Heinrich-Heine-University, Düsseldorf, Germany) [20]. 

For measurements of mechanical properties and DC, the normality of distribution was formally confirmed by Shapiro–Wilk’s test and normal Q–Q plots. Values of DC, MH, FS, and FM were compared using a three-way analysis of variance (ANOVA) with the factors: “*material*”, “*polymerisation protocol*”, and “*time*”. Considering the statistically significant interactions of the mentioned factors, for the “*material*” and “*polymerisation protocol*” factors, analysis was additionally performed by one-way ANOVA with Tukey’s post hoc correction for multiple comparisons. For the “*time*” factor, comparisons were made using the independent *t*-test observations assuming inhomogeneous variances.

Overall level of significance in all analyses was 0.05. Statistical analysis was performed using the SPSS software package (version 25; IBM, Armonk, NY, USA).

## 3. Results

### 3.1. Degree of Conversion

**Figure 2:**
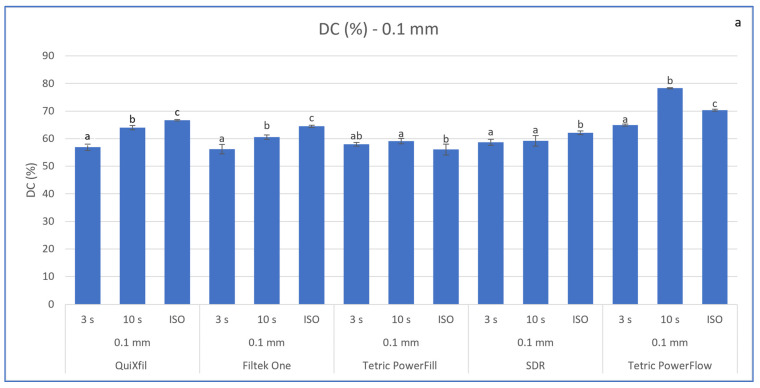

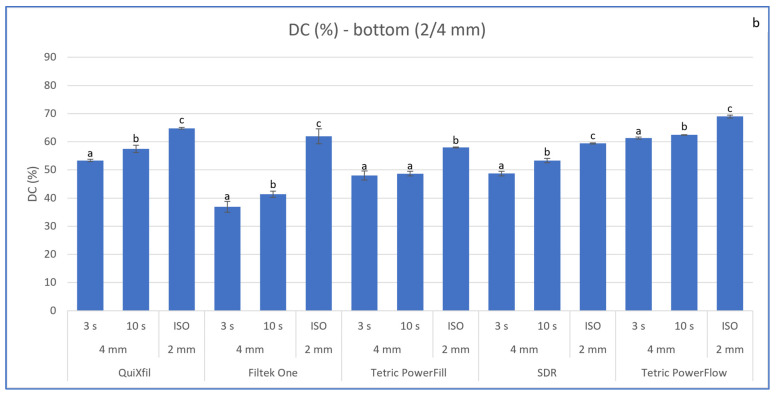
Comparison of mean DC (%) values (±SD) between polymerisation protocols within each time point for (**a**) 0.1 mm and (**b**) bottom at 2 or 4 mm. Identical letters indicate statistically homogeneous groups within the material.

Figure 2 shows a comparison of the DC values between the materials within each mode of polymerisation. The ISO protocol of polymerisation generally presented the highest DC values, except for Tetric PowerFlow, which reached the highest DC with the 10 s protocol. On the contrary, 3 s protocol presented the lowest DC results. The only material that demonstrated the same efficiency for the 3 s and 10 s protocols at 0.1 mm and 4 mm depth was Tetric PowerFill. Additionally, SDR did not show sensitivity to reduction in curing time at the 3 s protocol, but only at the superficial measurements at 0.1 mm. When comparing the DC at 0.1 or at a depth of 2 or 4 mm for the same material and curing regime, the DC was statistically higher at the superficial value of 0.1 mm than at a depth of 2 or 4 mm, except for Filtek One and Tetric PowerFill when cured for 20 s at 1000 mW/cm^2^.

### 3.2. Macromechanical Properties

**Figure 3:**
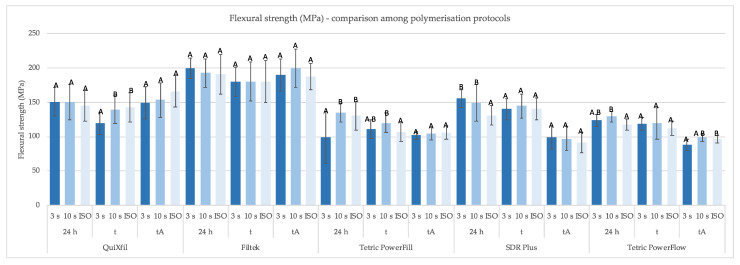
Mean values (±SD) of FS (MPa) comparing different polymerisation protocols in the same time points, separately for each material. Equal letters show statistically homogeneous groups for individual time points (*p* > 0.05); t: thermocycling, tA: thermocycling + absolute ethanol.

**Figure 4:**
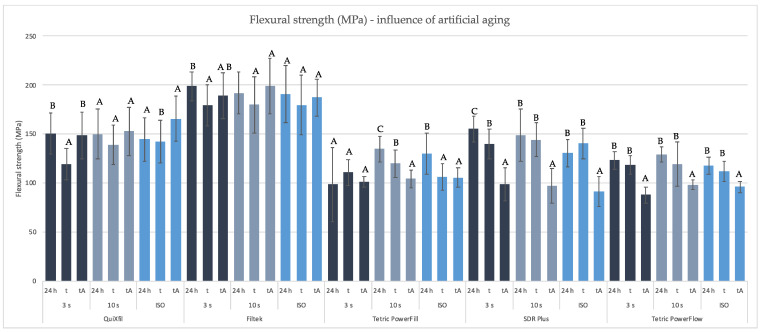
Mean values (±SD) of FS (MPa) comparing different time points within the same polymerisation protocol, separately for each material. Equal letters show statistically homogeneous groups for every individual method of polymerisation (*p* > 0.05); t: thermocycling, tA: thermocycling + absolute ethanol.

Figure 3 and Figure 4 show the FS values of all materials considering three time points and different polymerisation protocols. In general, Filtek One had the highest FS values, which were not affected by polymerisation protocols or artificial ageing. Figure 3 shows how the 3 s polymerisation protocol only had a negative effect on the FS of Tetric PowerFill after 24 h, but these differences were balanced out with the ISO protocol after thermocycling and thermocycling with exposure to absolute ethanol. In general, the FS after thermocycling with exposure to absolute ethanol, was statistically similar for all polymerisation protocols, except for Tetric PowerFlow.

Figure 4 shows a decrease in FS after thermocycling compared to the values measured after 24 h for most experimental groups. Exceptions are SDR (ISO protocol) and Tetric PowerFill (3 s protocol). It is interesting that some groups, such as QuiXfil (3 s protocol), show a higher FS after thermocycling and 7 days in absolute ethanol than after thermocycling.

**Figure 5:**
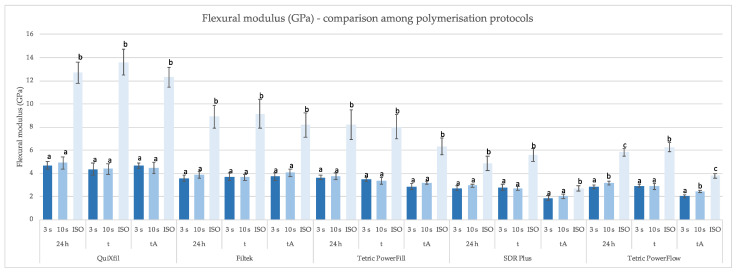
Mean values (±SD) of FM (GPa) comparing specimen preparation protocols within the same time points, separately for each material. Equal letters show statistically homogeneous groups for an individual time point (*p* > 0.05); t: thermocycling, tA: thermocycling + absolute ethanol.

**Figure 6:**
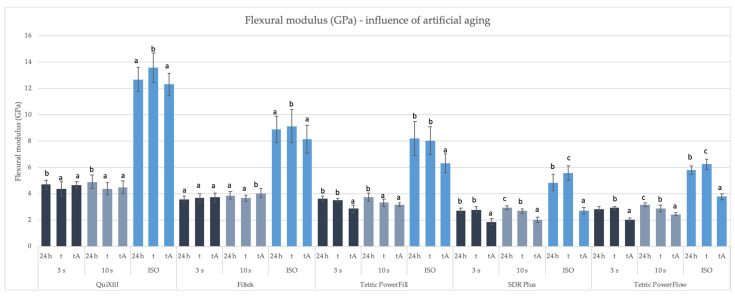
Mean values (±SD) of FM (GPa) comparing different time points within the same specimen preparation protocol, separately for each material. Equal letters show statistically homogeneous groups for each individual specimen preparation protocol (*p* > 0.05); t: thermocycling, tA: thermocycling + absolute ethanol.

Figure 5 and Figure 6 show the comparison of FM values between different time points and polymerisation methods. The values of the FM reached by ISO protocol were always higher than the 4 mm specimens polymerised by the 3 s and 10 s protocol. 

It is interesting to note in Figure 5 how specimens of all materials, with the exception of Tetric PowerFlow, polymerised with the 3 s and 10 s protocol, belonged to homogeneous groups within a defined time point, while the results of specimens polymerised by ISO standard were significantly higher.

Comparing each material individually in Figure 6, a non-uniform deterioration in FM after aging was observed. Some specimens did not show the expected deterioration of the FM after thermocycling. Moreover, their values were higher than the values of the same group measured after 24 h.

### 3.3. Micromechanical Properties

**Figure 7:**
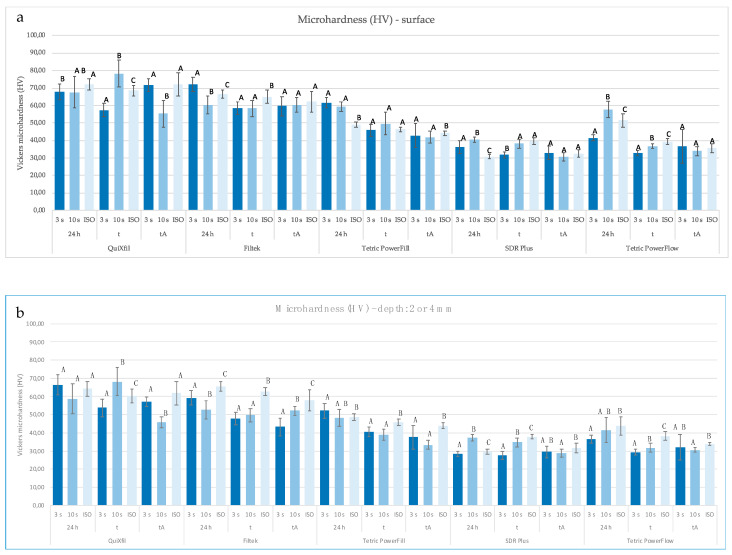
Mean values (±SD) of MH (HV) on (**a**) the surface and (**b**) the bottom of the specimen comparing different polymerisation protocols within the same time points, separately for each material. Equal letters show statistically homogeneous groups for each method of polymerisation (*p* > 0.05); t: thermocycling, tA: thermocycling + absolute ethanol.

**Figure 8:**
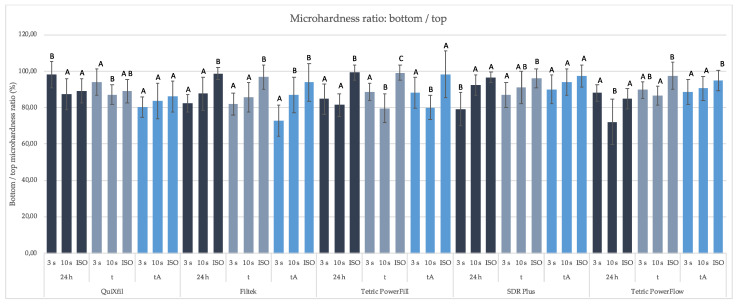
MH ratio (%) at 2/4 mm and on the surface (bottom/top ratio) in polymerisation within the same time points. Equal letters show statistically homogeneous groups for each method of polymerisation (*p* > 0.05); t: thermocycling, tA: thermocycling + absolute ethanol.

Comparing different polymerisation protocols within the same time points, the values of MH on the surface (Figure 7a) and on the bottom of the specimen (Figure 7b) are shown. Figure 8 shows the ratio of MH (%) on the bottom and on the surface (bottom/top ratio) of the specimen.

In Figure 7a,b, two of three high-viscosity bulk-fill composites (of which Filtek One on the lower surface, and QuiXfil on the upper surface) showed a reduction in the MH value due to polymerisation with the 3 s protocol, compared to the ISO protocol. The same effect applies to one of the two tested low-viscosity bulk-fill composites (Tetric PowerFlow), regardless of the surface of the specimen.

Figure 8 shows that all specimen preparation protocols produced more than 80% bottom/top MH ratio, except for Filtek One in 3 s protocol after thermocycling and ethanol and Tetric PowerFlow in 10 s protocol after 24 h.

## 4. Discussion

In this study, bulk-fill composites were evaluated by comparing the values of FS, FM, MH, and DC of specimens prepared according to the ISO protocol with the newly proposed testing of flexural strength and modulus using 4 mm specimens.

The main finding of this study is that FM can be significantly overestimated compared to 4 mm specimens when bulk-fill composite specimens are prepared according to the ISO 4049 standard. Therefore, the first null hypothesis is rejected. The FS and MH of the material depended mainly on the composition of the material and the proportion of filler particles, so the fourth null hypothesis was also rejected.

Certain materials exhibited a lower DC and poorer mechanical properties when polymerised for 3 s at high light intensity. However, the differences between the polymerisation protocols evened out after accelerated ageing in the most aggressive protocol—exposure to thermocycling, followed by degradation in absolute ethanol. Therefore, we can reject the second and third null hypotheses.

The DC is a fundamental property of any composite resin that affects most other properties, such as polymerisation shrinkage, macro- and micromechanical properties, monomer release, etc. [21]. By studying DC, the three polymerisation methods were compared in terms of their curing efficiency and the resulting changes in the micro- and macromechanical properties explained. When polymerising 4 mm specimens, an attempt was made to maintain “exposure reciprocity” and keep the total amount of energy approximately similar for both polymerisation protocols (3 s and 10 s). In the 3 s protocol, a large amount of energy is delivered quickly, while in the 10 s protocol, the energy is delivered more slowly. Due to the limitations of the polymerisation device, the 10 s polymerisation still delivered 2.2 J/cm^2^ more energy to the specimens than the 3 s polymerisation.

A number of papers challenged the concept of the exposure reciprocity [22]. In 2015, Selig et al. found that the exposure reciprocity is not valid for the values above 1500 mW/cm^2^ [23]. It was stated that the short exposure to high irradiance caused the rapid formation of numerous radicals and quick increase in viscosity as polymerisation progressed. This was the reason for the immobilisation of many unreacted monomers and the higher rate of bimolecular termination, resulting in a decrease in the final DC, which was about 70% compared to a longer cure with lower irradiances, as reported in the study by Selig et al. [23]. This was before the introduction of the “3 s materials” in 2019 [8]. The rapid curing of these materials is recommended by the manufacturer and explained by the RAFT polymerisation mechanism, i.e., the AFCT reagent β-allyl sulfone and the Norrish type I photoinitiator mentioned before [8,9,11,24]. In the case of Tetric PowerFill, the β-allyl sulfone is the reason for the formation of short-chain polymers and a higher mobility of the radicals, which lead to the equal DC values regardless of whether the energy was delivered to the material quickly or slowly [9,24,25].

The huge difference in the total amount of energy received by the 2 mm thick ISO specimens and the 4 mm thick specimens resulted in the highest DC for the ISO specimens. In the 3 s protocol, the 4 mm specimens received only 40% of the energy that ISO specimens received, and in the 10 s protocol, 50%.

Therefore, the 3 s protocol demonstrated a lower DC in comparison to the other two polymerisation methods for all tested materials, except for Tetric PowerFill. Only with Tetric PowerFill was DC statistically similar during 3 s and 10 s polymerisation, both at the surface and at 4 mm depth. This result is consistent with previous similar tests [25,26]. 

In contrast, the other material intended for 3 s polymerisation, Tetric PowerFlow, showed a better DC when polymerised with the 10 s protocol, but still achieved one of the highest DC values compared to the other materials tested here. Similar results were obtained in another study investigating the polymerisation kinetics of the same group of materials [27]. Tetric PowerFlow does not contain AFCT, but the high DC can be attributed to the lowest amount of fillers and close refractive indices of the organic matrix and the filler in the unpolymerised state, which are responsible for high translucency [8]. 

As expected, the DC values at the bottom of the specimen at 2 or 4 mm were lower than the DC of the same materials at 0.1 mm, which represents the near-surface value. The tops of the 2 and 4 mm specimens could not be used for surface DC measurements because it was not possible to establish close contact between the (already cured) specimen and the ATR crystal. Attempting to perform this in our preliminary study resulted in falsely low data for DC. However, the data from DC, obtained on a separate set of 0.1 mm samples, can be considered equivalent to the DC surface due to its small thickness. Bulk-fill composites transmit light better than conventional composites [6]. However, their light scattering is also subject to the Beer–Lambert law, according to which the light intensity decreases exponentially with increasing length of the light path [28]. Due to the attenuation of the light, polymerisation in deeper layers is therefore not achieved as well as at the surface. This was also confirmed in a previous study in which a transparent film was used at a depth of 2 mm as a separation between two specimen parts [4]. Ultimately, this also affects the mechanical properties.

In this study, 2 mm specimens polymerised according to the ISO standard had a significantly higher FM than 4 mm samples polymerised according to the 3 s or 10 s protocol. The ISO group of specimens had a generally higher DC, as these were polymerised on both sides and were only 2 mm thick, while the other two specimen groups were only polymerised on one side and were 4 mm thick. The part of the sample closer to the light source achieves a better cross-linking of the polymer network, while the lower layers probably achieve an increasingly more linear polymer structure due to light attenuation [29]. For this reason, the cured composites do not have a homogeneous structure, but are characterised by microregions of a lower polymerised network enveloping each filler particle [30]. Although the study of the cross-linking of the polymer network was not the subject of this research, it is known that specimens with a thickness of 4 mm show a polymerisation gradient from the light source to the deeper layers [21].

It could be concluded that the ISO samples likely had a more densely cross-linked polymer than the 4 mm samples, contributing to a higher stiffness and FM. This hypothesis was also confirmed in the earlier study with separate top and bottom 2 mm specimens, where the bottom 2 mm specimens had a much lower FM than the upper ones [4]. By transferring these results to the current study, it can be assumed that the lower parts of the 4 mm specimens had a higher elasticity (lower FM), which allowed for a higher bending in the three-point bending test. The greater elasticity of the 4 mm specimens resulted in a delayed breaking point of the specimen, which is why their final FS values matched those of the ISO protocol.

As expected, FM in our study was the highest for QuiXfil and decreased towards materials with lower filler contents, namely SDR and Tetric PowerFlow. Masouras et al. [31] pointed out that the amount of filler is the most important factor for FM, while the shape and size of the filler play a minor role [32,33,34]. In addition to the filler content, Randolph et al. [35] found that materials with pre-polymerised particles had lower FM values compared to the other materials tested. Comparing the values of FM of high viscosity materials, Filtek One shows higher values than Tetric PowerFill. The composition of Filtek One features solid zirconium and silica nanoparticles, unlike Tetric PowerFill, which contains pre-polymerised filler particles consisting of an organic matrix with incorporated a glass microfiller. Pre-polymerised particles contribute to stress reduction but do not have a significant impact on improving mechanical properties. This is probably the reason why Filtek One has higher FM values than Tetric PowerFill [36].

SDR shows interesting behaviour under three-point loading. The SDR specimens showed a notable deformation under force before they fractured. The values of FS were higher than, for example, the results of Tetric PowerFlow and Tetric PowerFill. The organic component of the SDR—primarily high-molecular weight-modified UDMA—likely increased the flexibility of the material, as evidenced by the lowest values of FM among all tested materials compared to different polymerisation protocols. The same was shown in a previous study [4].

There was generally no difference in FM values of specimens polymerised with 3 s and 10 s protocols. The only statistically significant difference was observed with Tetric PowerFlow when measured after 24 h and after thermocycling, followed by ethanol immersion. For the FS values, the comparison of the two protocols within each material showed a statistically significant difference for QuiXfil (measured after thermocycling) and Tetric PowerFill (measured after 24 h). Therefore, most results were statistically similar.

Tetric PowerFill has a similar DC with both the 3 s and 10 s protocols, so the difference in FS after 24 h can be attributed to the higher heterogeneity of the polymer network at the bottom of the 3 s specimens [37]. These differences were equalised after thermocycling. It is likely that exposure to high temperatures during thermocycling mobilised the remaining unreacted radicals and improved post-cure polymerisation, and thus, FS.

On the other hand, QuiXfil had a much lower DC with the 3 s protocol than with the 10 s protocol, which explains the lower FS for 3 s curing. In the case of the highly filled QuiXfil, the mobility of unreacted species was probably hindered by the fillers, resulting in a lower DC and underlining the poorer mechanical performance of this material with the 3 s protocol.

Under ideal conditions, composites should remain stable and unchanged, but conditions in the oral cavity are extremely challenging and it is necessary to conduct studies that involve the artificial ageing of the material. The thermocycling procedure performed in this study (10,000 cycles with temperature changes from 5 to 55 °C and a duration of 30 s each) is considered to simulate the temperature changes that occur in the oral cavity over the course of one year, i.e., 20–50 cycles per day [14].

A separate experimental group was additionally aged in absolute ethanol for 7 days after thermocycling to further enhance hydrolytic degradation. Ethanol, as a strong solvent, penetrates more easily into the less cross-linked polymer network, separating the physical bonds (e.g., van der Waals forces) between the chains and thus weakening the structure of the organic matrix. Therefore, such materials are more prone to the weakening of the mechanical properties in ethanol than those with more dense chemical cross-links between the chains that are not affected by ethanol [37].

The results show that the process of thermocycling with exposure to ethanol resulted in more degradation for materials with a higher organic matrix content and with pre-polymerised particles. In addition, 3 s polymerisation resulted in a greater degradation of FM when exposed to ethanol, which was mostly not observed for samples subjected to thermocycling only. This finding, together with the confirmation provided by results of MH, indicates the formation of a more linear polymer structure at the bottom of the 4 mm specimens. This was also confirmed in a study by Graf and Ilie in 2020, in which only one material was tested, Tetric PowerFill [37].

Despite the aforementioned considerations, it is important to emphasise that even the harshest conditions in this study did not cause FS to drop below 80 MPa, which was postulated as a minimum by ISO [18]. All materials tested proved satisfactory for clinical use under all polymerisation protocols and under the most severe accelerated ageing conditions.

In summary, FM and FS are clinically relevant parameters as they represent the material’s ability to resist deformation and fracture. Heinze et al. found in a meta-analysis a significant correlation between the decrease in FS after ageing in ethanol and the clinical index [38], which underlines the value of our results for clinical practice. The decrease in FM in the 4 mm samples found in this study is also significant. While a high FM is important to be as similar as possible to dentin values, a low FM is desirable to minimise the negative consequences of stress caused by polymerisation shrinkage [39]. The reduced FM in the 4 mm layers of bulk-fill composites could contribute to the good clinical performance of these materials by partially relieving shrinkage stresses.

The MH results suggest that differences in material composition were a more important source of variability in micromechanical properties than curing protocol and that the effects of changing curing parameters were material-dependent. The filler phase had the strongest effect on the MH. This confirms the conclusions of most other studies that filler amount is the dominant factor [40,41,42].

The MH values resulting from different polymerisation methods were mostly significantly different with respect to the corresponding ageing method and differed between materials. A longer polymerisation time primarily led to an improvement in the micromechanical properties. When polymerising with the ISO protocol, the highest MH values were obtained on both the upper and lower surfaces of the tested samples. Additionally, after thermocycling, the group with the ISO protocol generally achieved the highest MH values.

The bottom-to-top MH ratio of 80% has traditionally been used as an arbitrary threshold for the quality of the curing of composite specimens [41,43]. In our study, suboptimal curing efficiency was found for Tetric PowerFlow (24 h) with a 10 s protocol and for Filtek One (after tA) with a 3 s protocol. Both materials achieved a bottom-to-top MH ratio of 72%. Apart from this, the other bulk-fill composites tested showed an adequate curing efficiency regardless of the polymerisation protocol used. As shown here and in our previous study [4], the 3 s curing protocol seems not to be appropriate for Filtek One, which is probably due to the long, high-molecular-weight AUDMA monomer and high filler content, which limit the mobility of radical species. The manufacturer recommends three repetitions of 20 s cure cycle from all sides of the restoration for the use of this material in class II cavities, which seems justified. On the other hand, the low bottom-to-top MH ratio of Tetric PowerFlow is due to the very high surface MH values for the 10 s and ISO curing, which were above the average of the low-viscosity materials at all time points. The MH values are in accordance with the highest DC value at 0.1 mm for the same protocols. In contrast, MH and DC were significantly lower at 4 mm depth, resulting in the low bottom-to-top MH ratio.

## 5. Conclusions

This in vitro study showed that ISO 4049 may not be suitable for testing the macromechanical properties of bulk-fill composites, as unrealistically high values of FM are obtained for 2 mm specimens. An alternative method is proposed using 4 mm thick specimens together with one-sided polymerisation.

High-power ultrafast polymerisation resulted in lower values of DC, with the exception of Tetric PowerFill. FS and MH were mainly influenced by the type of material and filler content. After an accelerated ageing induced material degradation, there was no significant difference in FS, regardless of the polymerisation method. Within the limits of the current study, ultrashort polymerisation for Tetric PowerFill could be acceptable for clinical work, especially considering the reduced chair time, the possibility of errors, and the compensation of differences between the polymerisation methods due to artificial aging.

## Figures and Tables

**Table 1:** Composition of the investigated materials according to the manufacturer’s instructions.

Viscosity	Material (Abbreviation) Manufacturer	Organic Matrix	Fillers (wt%/vol%)
**High viscosity**	QuiXfil^®^ Posterior Restorative (QXL) Dentsply Sirona; Charlotte, NC, USA	Bis-EMA, UDMA, TEGDMA, dimethacrylate and trimethacrylate resin, carboxylic acid modified dimethacrylate resin	silanised aluminium–sodium–fluorine–phosphate glass (86/66)
**High viscosity**	3M™ Filtek™ One Bulk Fill Restorative (FIL) 3M ESPE Dental Products; St. Paul, MN, USA	AUDMA, diurethane-DMA, 1,12-dodecan-DMA	non-aggl./non-aggr. silica, non-aggl./non-aggr. zirconia, aggr. zirconia/silica cluster, aggl. ytterbium trifluoride (~76.5/~58.5)
**High viscosity**	Tetric^®^ PowerFill (PFL) Ivoclar Vivadent AG; Schaan, Liechtenstein	monomer matrix—dimethacrylate (wt = 20–21%)	barium glass, ytterbium trifluoride, mixed oxide, copolymers (76–77/53–54)
**Low viscosity**	SDR^®^ Plus Bulk Fill Flowable (SDR) Dentsply DeTrey GmbH; Konstanz, Germany	resin matrix—modified UDMA, TEGDMA, dimethacrylate and three methacrylate resins	silanised barium–aluminium-fluoro-borosilicate glass, silanised strontium aluminium–fluoro-silicate glass, surface treated silica, ytterbium fluoride, synthetic inorganic iron oxide pigments, titanium dioxide (70.5/47.4)
**Low-viscosity**	Tetric^®^ PowerFlow (PFW) Ivoclar Vivadent AG; Schaan, Liechtenstein	monomer matrix—dimethacrylate (wt = 28%)	barium glass, ytterbium-trifluoride, copolymers (68.2/46.4)

Bis-EMA: ethoxylated bisphenol A dimethacrylate; UDMA: urethane dimethacrylate; TEGDMA: triethylene glycol dimethacrylate; AUDMA: aromatic urethane dimethacrylate; DMA: dimethacrylate; non-aggl.—non-agglomerated; non-aggr.—non-aggregated; aggl.—agglomerated; aggr.—aggregated.

## Data Availability

The data that support the findings of this study are available from the corresponding author, D.M., upon reasonable request.
